# Dramatic In Vivo Efficacy of the EZH2-Inhibitor Tazemetostat in *PBRM1*-Mutated Human Chordoma Xenograft

**DOI:** 10.3390/cancers14061486

**Published:** 2022-03-14

**Authors:** Thibault Passeri, Ahmed Dahmani, Julien Masliah-Planchon, Adnan Naguez, Marine Michou, Rania El Botty, Sophie Vacher, Rachida Bouarich, André Nicolas, Marc Polivka, Coralie Franck, Anne Schnitzler, Fariba Némati, Sergio Roman-Roman, Franck Bourdeaut, Homa Adle-Biassette, Hamid Mammar, Sébastien Froelich, Ivan Bièche, Didier Decaudin

**Affiliations:** 1 Laboratory of Preclinical Investigation, Translational Research Department, Institut Curie, University of Paris Saclay, 75005 Paris, France; thibault.passeri@neurochirurgie.fr (T.P.); ahmed.dahmani@curie.fr (A.D.); naguezadnan@gmail.com (A.N.); marine.michou@gmail.com (M.M.); rania.el-botty@curie.fr (R.E.B.); fariba.nemati@curie.fr (F.N.); 2 Department of Genetics, Institut Curie, University of Paris Saclay, 75005 Paris, France; julien.masliahplanchon@curie.fr (J.M.-P.); sophie.vacher@curie.fr (S.V.); coralie.franck@curie.fr (C.F.); anne.schnitzler@curie.fr (A.S.); ivan.bieche@curie.fr (I.B.); 3 Department of Neurosurgery, Lariboisière Hospital, Assistance Publique des Hôpitaux de Paris, University of Paris, 75010 Paris, France; sebastien.froelich@aphp.fr; 4 Integrated Cancer Research Site, Institut Curie, 75005 Paris, France; rachida.bouarich@curie.fr (R.B.); franck.bourdeaut@curie.fr (F.B.); 5 Department of Tumor Biology, Institut Curie, 75005 Paris, France; andre.nicolas@curie.fr; 6 Department of Pathology, Lariboisière Hospital, Assistance Publique des Hôpitaux de Paris, University of Paris, UMR 1141 Inserm, 75010 Paris, France; marc.polivka@aphp.fr (M.P.); homa.adle@aphp.fr (H.A.-B.); 7 Department of Translational Research, Institut Curie, University of Paris Saclay, 75005 Paris, France; sergio.roman-roman@curie.fr; 8 Proton Therapy Center, Institut Curie, 91400 Orsay, France; hamid.mammar@curie.fr; 9 Department of Medical Oncology, Institut Curie, 75005 Paris, France

**Keywords:** chordoma, patient-derived xenografts, next-generation sequencing, EZH2 inhibitor, targeted therapy

## Abstract

**Simple Summary:**

Chordomas are rare bone tumors characterized by a high recurrence rate. Presently, no medical treatment is available for advanced diseases due to the lack of molecular data and preclinical models. The current study showed the establishment and characterization of the largest panel chordoma xenografts, allowing pharmacological studies. In one *PBRM1*-mutated model, we demonstrated a strong therapeutic efficacy of the EZH2-inhibitor tazemetostat, encouraging further research on EZH2-inhibitors in chordomas.

**Abstract:**

Chordomas are rare neoplasms characterized by a high recurrence rate and a poor long-term prognosis. Considering their chemo-/radio-resistance, alternative treatment strategies are strongly required, but their development is limited by the paucity of relevant preclinical models. Mutations affecting genes of the SWI/SNF complexes are frequently found in chordomas, suggesting a potential therapeutic effect of epigenetic regulators in this pathology. Twelve PDX models were established and characterized on histological and biomolecular features. Patients whose tumors were able to grow into mice had a statistically significant lower progression-free survival than those whose tumors did not grow after in vivo transplantation (*p* = 0.007). All PDXs maintained the same histopathological features as patients’ tumors. Homozygous deletions of *CDKN2A/2B* (58.3%) and *PBRM1* (25%) variants were the most common genomic alterations found. In the tazemetostat treated PDX model harboring a *PBRM1* variant, an overall survival of 100% was observed. Our panel of chordoma PDXs represents a useful preclinical tool for both pharmacologic and biological assessments. The first demonstration of a high antitumor activity of tazemetostat in a PDX model harboring a *PBRM1* variant supports further evaluation for EZH2-inhibitors in this subgroup of chordomas.

## 1. Introduction

Chordomas are rare bone neoplasms derived from notochordal cell rests. Their origin explains their most common location in the midline neuraxis [1]. They are considered to be slow growing; however, recurrence rates are high, and the long-term prognosis remains poor. The global survival outcome is 65% and 32% at 5 and 10 years, respectively [2]. The mainstay therapeutic strategy consists of a maximal surgical resection followed by a high-dose proton therapy [3,4]. Considering their close relationship with eloquent neurological structures, maximal resection could be difficult to achieve, which is especially problematic given the chemoradiotherapy resistance of chordomas [5]. Thus, alternative treatment strategies are strongly required because of the restricted efficacy of surgery and/or reirradiation in the relapse tumor [3].

Presently, no medical adjuvant therapy is available for inoperable or advanced diseases unmanageable with surgery and/or radiotherapy. Moreover, the development of therapeutic options is limited by the paucity of relevant preclinical models. The growing knowledge about the mutational profile of chordomas [6,7] involved in oncogenesis and tumor progression has led to the identification of new therapeutic targets and emerging therapies. For such a rare disease, testing the efficacy of these innovative therapies in clinical trial remains difficult. To better characterize the chordoma physiopathology and to understand the tumor response to select therapeutics agents, preclinical animal models are mandatory before the assessment of innovative treatments in human clinical trials. Xenografts, derived from primary patient tumors, are well known as unmissable preclinical models to test therapeutic agents in the field of oncology, reproducing the heterogeneity of human cancer and retaining the genotypic and phenotypic features of the original human tumor, as well as the response to therapy [8,9,10,11,12]. In the past, several chordoma models have been established and grouped: cell lines [13,14,15,16,17,18], cell line-derived xenografts [14,18,19,20,21,22], heterotopic primary xenografts [10,23,24,25] and orthotopic primary xenografts [11,12]. However, these xenografts were often unique, derived from one chordoma location, and in vivo pharmacologic studies on these models remain scarce.

The Switch/Sucrose Non-Fermentable (SWI/SNF) complexes, which are a family of ATP-dependent chromatin remodeling complexes playing critical roles in controlling gene transcription and DNA repair, are altered in approximately 20% of cancers [26]. Mutations in subunits of SWI/SNF complexes, including the *PBRM1* gene, have frequently been reported in chordomas as cancer driver genes [6,7]. Those mutations lead to an upregulation of the enhancer of zeste homolog 2 (EZH2) activity, a subunit of Polycomb repressive complex 2 (PRC2), known as a tumor driver and thus a potential therapeutic target in chordomas [27]. Therapeutic preclinical and clinical studies [27,28] in cancers harboring mutations in the SWI/SNF complex subunits have recently demonstrated promising results, highlighting the fact that epigenetic regulators, such as EZH2-inhibitors, appear as a hot research topic. Hence, the *in vitro* targeted therapeutic effect in *PBRM1*-deficient clear cell renal cell carcinoma (ccRCC) has recently been described, suggesting a possible similar activity in *PBRM1*-mutated chordoma [29].

In this study, we established and well characterized a panel of primary human chordoma xenografts from patients’ samples transplanted into immunodeficiency nude mice. Then, we demonstrated a strong therapeutic efficacy of the EZH2-inhibitor tazemetostat (E7438/EPZ6438) in one of our PDX models harboring a *PBRM1* mutation.

## 2. Materials and Methods

### 2.1. Patients and Tumor Samples

Tumor samples were obtained from 38 patients with confirmed diagnosis of chordoma on pathological analysis; those tumor samples were collected between March 2015 and March 2018. All patients provided written consent for future experimental research for academic purposes at the time of treatment, including histopathological and genetic analyses. Tumor samples were directly obtained from the operating room of the neurosurgical department of Lariboisière Hospital (Paris, France) and immediately transferred to the Laboratory of the Pre-clinical Investigation (Institut Curie, Paris, France). Some samples were also fixed in acetic acid for further immunohistochemical and morphometric analyses and/or collected in liquid nitrogen for pangenomic analyses.

### 2.2. Data Collection

For each patient, all useful clinical data were collected: age, sex, tumor radiological characteristics (volume, location, intradural invasion), previous surgery and/or radiation for chordoma and follow-up (months). Extent of resection (EOR) was determined by an expert neuroradiologist on the MRI obtained within 48 h after surgery, and was classified as gross total (GTR, no residual tumor) or not, as it is usually recommended and performed in clinical practice [30]. Follow-up data included tumor recurrence and metastasis occurrence. Progression-free survival (PFS) was defined as the time from date of surgery to the time of the first tumor progression (tumor recurrence or metastasis occurrence).

### 2.3. Establishment of Chordoma Heterotopic (Subcutaneous) Xenografts

All in vivo experimental procedures, animal care and housing were performed in accordance with the recommendations of the European Community (2010/63/UE) for the care and use of laboratory animals. Experimental procedures were specifically approved by the ethics committee of the Institut Curie CEEA-IC No.118 (authorization APAFiS No. 25870-2020060410487032-v1 given by the national authority) in compliance with the international guidelines. The heterotopic subcutaneous implantation was chosen considering the simplicity of the surgical technique and the ease of follow-up with the mice. For the first passage (P0), nude mice were anesthetized with xylazine/ketamine anesthesia. Next, after a skin incision, a fresh tumor sample (3–4 mm diameter) was carefully transplanted into the interscapular fat pad of 2 to 4 immunodeficiency nude female mice. The skin was closed with staples. Following the surgery, the mice were observed at 37 °C, under heat lamp, until awakening. Swiss nude mice, 5 to 7-week-old-females, were purchased from Charles River laboratories (Les Arbresles, France). All mice were then maintained in a specific pathogen-free animal house and regularly observed for tumor growth. Mice without growing tumors one year after initial transplantation were sacrificed. At a volume of approximately 1 cm^3^, tumors were removed and subsequently transplanted to naive nude mice. Samples were concomitantly stored frozen in DMSO-FCS solution or directly in liquid nitrogen and fixed in formol for further studies. After three consecutive mouse-to-mouse passages, the xenograft was considered stabilized and was submitted to the process for extensive characterization.

At the end of characterization, xenografts were considered as validated for any further in vivo pharmacological assessment.

### 2.4. Histopathological and Immunohistochemical Analyses

Morphologic examination was realized on each xenograft and compared with the histologic features of the corresponding patient’s tumor. For light microscopic examination, 5-µm-thick formalin-fixed-paraffin-embedded sections were stained with hematoxylin, eosin, and saffron (HES), Alcian blue and processed for immunohistochemistry (IHC) using antibodies against brachyury, cytokeratin (CK) AE1/AE3, and Ki-67. Tumors were classified according to the WHO classification 2016 (classic, chondroid, and dedifferentiated subgroups). The Ki-67 labeling index (LI) was calculated using the ImageJ “cell counter” tool (https://imagej.nih.gov/ij/ accessed on 12 December 2020) on photographs obtained at ×400 magnification. H3K27me3 staining was assessed in each xenograft model using the Tri-Methyl-Histone H3 (Lys27) (C36B11) Rabbit mAb (ref: 9733; Cell Signaling) at 1/1000 for an incubation time of 60 min at room temperature after unmasking the tissue for 20 min at pH 6.

### 2.5. Biomolecular Analysis

DNA was extracted from frozen tumor samples (xenograft and the patient’s tumor) using a standard phenol/chloroform procedure.

A total of 50 ng of genomic DNA extracted from the tumor derived from xenografts and a part of the patient’s tumors were analyzed for protein-coding gene mutations, by targeted next-generation sequencing (NGS). The in-house NGS panel includes 571 genes of interest in oncology for diagnosis, prognosis and theranostics, including chordomas genes of interest [6], such as *PIK3CA*, *PTEN*, *CDKN2A/2B*, *PBRM1*, *SETD2* and *ARID1A*. The library preparation was performed using the Agilent Sureselect XT HS kit, and sequencing was completed on an Illumina NovaSeq 6000 sequencer. All variants, using Varscan2 (v2.4.3-0), that passed the following thresholds were validated: allelic ratio above 5% and population frequency lower than 0.1% in 1000 g, ESP or gnomAD. This large targeted NGS panel also allowed molecular analysis of tumors for CNV (copy number variation) using the design of the 571 genes and an additional backbone of probes across the whole genome with an average resolution of 1 probe every 200 Kb. This allows for the determination of the ploidy and an estimated cellularity, together with a genomic profile spanning every chromosome. The copy number profile for each tumor was estimated using a combination of homemade R scripts and a facets package (v0.6.0) with a sex-specific unmatched-germline control previously sequenced using the same panel for normalization. In case of doubt concerning the originating link between the patient’s tumors and PDXs, an identity monitoring was performed based on polymorphisms sequenced by the panel.

### 2.6. Antitumor Efficacy of the EZH2-Inhibitor Tazemetostat

Our pharmacological study focused on one PDX harboring a *PBRM1* mutation. Hence, the in vivo pharmacologic experiment was performed to assess the activity of the EZH2 inhibitor, tazemetostat (Tazverik^®^, Epizyme, Cambridge, MA, USA). Tazemetostat was administered orally at a dose of 75 mg/kg twice a day, 5 days per week, representing an optimal dosing schedule adapted in human patients (800 mg twice a day). The treatment was administered from day 1 to mouse sacrifice.

For in vivo therapeutic studies, a 15 mm^3^ tumor fragment was grafted subcutaneously into 30 female immunodeficient nude mice. Mice bearing growing tumors with a volume of 60 to 150 mm^3^ were randomly assigned to control or treatment groups. Animals with tumor volumes outside this range were excluded. Treatments were started on day 1. Mice were weighted and tumors were measured once a week. Xenografted mice were sacrificed when the tumor volume reached 430 mm^3^, considering the slow growth of these tumors.

Tumor volumes (in mm^3^) were calculated using two perpendicular diameters with calipers as the following: V (volume) = (a × b)^2^/2 where a and b are the largest and smallest perpendicular tumor diameters (in mm). Relative tumor volumes (RTV) were calculated from the following formula: RTV = (Vx/V1), where Vx is the tumor volume on day x and V1 is the tumor volume at initiation of therapy (day 1). Antitumor activity was evaluated according to tumor growth inhibition (TGI), which was calculated according to the following formula: percent GI = 100 − (RTVt/RTVc ×100), where RTVt is the median RTV of treated mice and RTVc is the median RTV of controls, both at a given time point when the antitumor effect was optimal. A meaningful biological effect was defined as a TGI of at least 50%. Statistical significance of differences observed between the individual RTVs corresponding to the treated mice and control groups was calculated by the two-tailed Mann–Whitney test. Growth delay index was calculated as the time required to reach the same RTV in the treated and control groups, at an RTV of 2.

Moreover, an overall response rate (ORR) was calculated for each treated mouse as follows: [(RTVt/mRTVc)], where RTVt is the relative tumor volume of the treated mouse and mRTVc is the median relative tumor volume of the corresponding control group at the end of treatment. We then calculated [(RTVV)-1] for each treated mouse. A tumor was considered to be responding to treatment if [(RTVV)-1] was below −0.5.

### 2.7. Pharmacodynamics Study after In Vivo Pharmacological Experiments

At the end of the tazemetostat treatment, we collected fresh tumors and peripheral blood mononuclear cells (PBMC) to assess the efficacy of the drug on EZH2 activity. PBMCs were exposed to RBC lysis buffer (NH_4_Cl: 155 mM; NaHCO_3_: 10 mM; EDTA: 0.1 mM) for 5 min on ice and washed with MACS buffer twice to remove red blood cells. Subsequently, PBMC were labeled with Aqua dead (L34966A; Invitrogen) for 30 min at 4 °C in PBS and subsequently fixed with fixation/permeabilization buffer (Ref: 00-5223-56, Invitrogen) during 30 min at 4 °C. Afterward, cells were intracellularly labeled with histone H3 antibody (ref: 12230S; Cell Signaling) and the specific H3K27me3 antibody (reference: 5499S) for 30 min at 4 °C in permeabilization buffer (Ref: 00-8333-56, Invitrogen). Finally, cells were washed and resuspended in MACs buffer for FACS analysis in BD LSR Fortessa. Gates were defined on single and live cells and the signal of H3K27me3 staining was calculated as a ratio of total H3 staining. In parallel, freshly resected tumors were fixed in formol/ethanol, and then embedded in paraffin; H3K27me3 staining was assessed using the tri-methyl-histone H3 (Lys27) (C36B11) with the previously described procedure. Brain delivery was not evaluated considering the extradural bone origin of chordomas.

### 2.8. Statistical Analyses

Statistical analyses were performed using the Prism v9.0 software (GraphPad Software, Inc., La Jolla, CA, USA) and R studio software version 1.4. Statistical characteristics were used to describe all variables. Numerical variables were expressed by the median or mean and standard deviation as appropriate. Categorical variables were expressed as the count and percentage. Variables were tested by the Mann–Whitney test, Fisher’s test or Chi2 test as appropriate. PFS was determined using the Kaplan–Meier method and the log rank (Mantel–Cox) or Wilcoxon test were used to compare groups. A multivariate Cox proportional hazards model was also used to test the differences across the in vivo tumor in the mice with the adjustment of all criteria for which PFS was statistically significant in univariate analysis, i.e., Ki-67 LI and GTR.

Every statistical test used in this manuscript was two-tailed and *p*-values lower than 0.05 were considered as significant results.

## 3. Results

### 3.1. Establishment of Xenografts

A total of 38 chordoma samples obtained from primary tumors were implanted into nude mice. Among the 38 tumors transplanted, 12 gave rise to viable tumors (after three consecutive mouse-to-mouse passages (P3), take rate: 31.6%). Among them, two xenografts (CD36, CD44) were sacrificed before P2 (Covid19 pandemic period) but after reimplantation, a tumor growing on one passage was noted. We observed a slow growth of all new models, corresponding to the natural tumor’s speed observed in the clinics. Hence, histological, molecular and genetic characterization was performed on these 12 models.

Clinical characteristics of the 38 patients, as well as their possible impact on the in vivo growth of the corresponding xenografted tumors, are summarized in Table 1. The overall mean follow-up with patients was 37.2 ± 12.1 months (range: 7.6–57; median: 39.7). A total of 21 patients (55.3%) had already been operated on and/or irradiated before being referred to our institution. The tumor take rate was significantly increased when the primary tumor volume was superior to 30 cm^3^ (*p* = 0.03). Tumor take was also higher when the gross total resection was not achieved during surgery (*p* = 0.002). A significant correlation between the in vivo tumor take and a high level of Ki-67 LI was noted (*p* = 0.001), but not with histological types (*p* = 0.53). The percentage of tumor graft seemed to be higher, without reaching significance, when the primary tumor was intradural (*p* = 0.058). No significant correlation was found between the percentage of tumor take and the tumor location (*p* = 0.48) nor in case of tumor relapse after previous surgery and/or radiation (*p* = 0.16).

**Figure 1:**
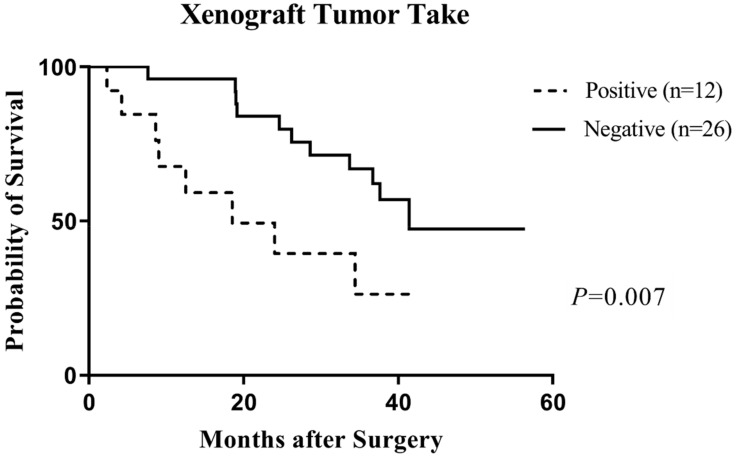
Prognostic value of the in vivo tumor take on the progression-free survival (PFS) of corresponding chordoma patients (univariate analysis). The PFS was significantly lower for patients whose tumors were able to grow into nude mice compared to patients whose tumors did not grow after in vivo transplantation (Wilcoxon test, *p* = 0.007).

During the follow-up period, 19 patients presented a recurrence (50%). The PFS ranged between 2.3 and 56.4 months (mean: 28.9 ± 13.8, median: 3.9). Relevant patients’ tumor features (previous treatment, Ki-67 LI, GTR, location), as well as the capacity of the tumor to grow into mice, were tested for their impact on patients’ PFS. The median PFS was 18.5 months for patients whose tumors were able to grow into mice and 83.9 months for patients whose tumors did not growth after in vivo transplantation (*p* = 0.007) (Figure 1). Log rank statistics also revealed statistically significant differences in terms of PFS for Ki-67 LI ≥ 6% and GTR (*p* = 0.03 and *p* = 0.01, respectively) but not with previous treatment (*p* = 0.16) or location (*p* = 0.49). Next, for the capacity of the tumor to grow into mice, Ki-67 LI and GTR were entered into a multivariate Cox hazards model to determine if they were independently predictive of PFS. None of these parameters was independently predictive of PFS (tumor take, *p* = 0.34; GTR, *p* = 0.19; Ki-67 LI, *p* = 0.32).

Interestingly, a correlation was supposed, without reaching significance, between in vivo tumor growth and the metastasis development in patients (*p* = 0.051).

### 3.2. Histopathological Analysis

**Figure 2:**
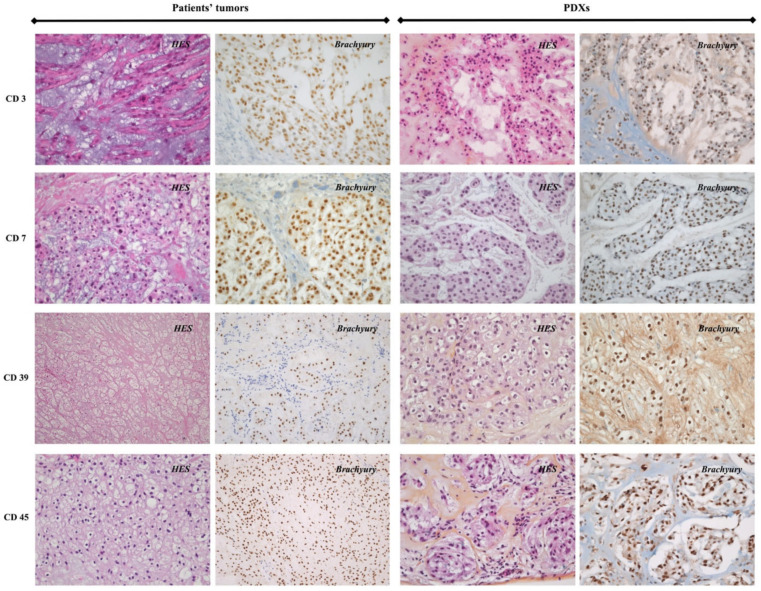
Histological and immunohistochemical comparison between primary patients’ tumors and their corresponding xenografts (CD3, CD7, CD39 and CD45 models). Histological analyses with HES showed that xenografts resembled the primary tumors from which they derived. All primary patients’ tumors and their corresponding xenografts were immunolabeled with brachyury (magnification ×400).

Results of the histopathological analysis between the primary patients’ tumors and their corresponding xenografts are shown in Table 2. Histological analyses showed that xenografts resembled the primary tumors from which they derived, e.g., classic chordoma comprising physaliphorous cells on a myxoid matrix, and chondroid chordoma comprising chondroid matrix on some states (Figure 2). Classic chordoma (91.7%) was the most common type observed, followed by the chondroid type (8.3%). All primary patients’ tumors and their corresponding xenografts were immunolabeled with brachyury and CK AE1/AE3, which were positive in all studied tumors. Concerning the Ki-67 LI, according to the grading of chordomas as previously reported [31], all xenografts harboring a Ki-67 LI ≥6% or <6% were derived from primary patients’ tumors with a Ki-67 LI ≥6% or <6%, respectively.

**Figure 3:**
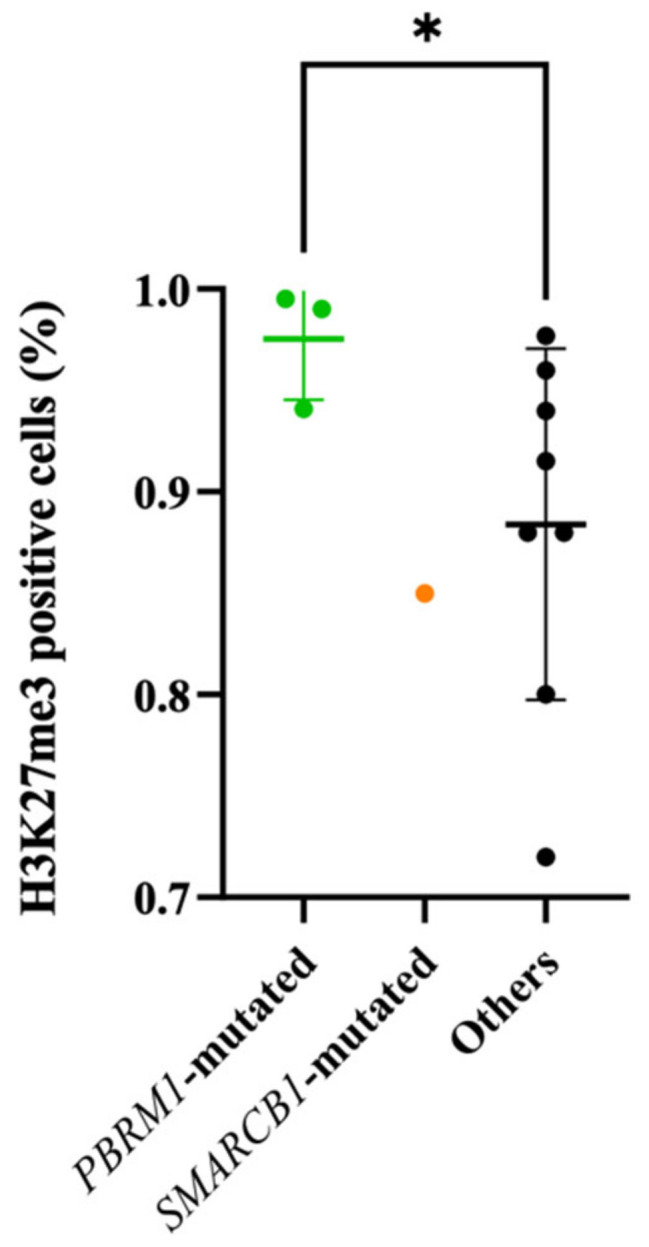
Percentage of H3K27me3 positive cells according to *PBRM1*-mutated status in the 12 PDX chordoma models. A significantly higher expression of H3K27me3 in the *PBRM1*-mutated compared to non-*PBRM1*-mutated PDX models was noted. * achieve statistical significance compared to control (*p* < 0.05) by Mann–Whitney test.

In parallel, we compared the expression level of H3K27me3 between *PBRM1*-mutated models and those that were not (Figure 3). We observed a significantly higher expression level of H3K27me3 in the three *PBRM1*-mutated PDX models (*p* = 0.05).

### 3.3. Genomic Analysis

All 12 xenografts were analyzed on the NGS panel. The genomic alterations of all the xenografts with their standard mutation nomenclature are represented in the Table 2. Five primary tumors, including the one corresponding to the model tested in the pharmacologic program (i.e., CD39), were also analyzed to compare patients’ tumors and the corresponding xenografts.

On the 12 models, the homozygous deletion of *CDKN2A/2B* was the most common genomic alteration found by the CNV analysis (58.3%). Inactivating pathogenic variants affecting the SWI/SNF complex were observed: *PBRM1* (c.3222del, c.599C>G, c.2377C>T, 25%) and *SMARCB1* (c.93+2T>G, 8.3%). Mutations in the SWI/SNF complexes were mutually exclusive with homozygous deletions of *CDKN2A/2B* in our panel (*p* = 0.008).

Four other variants were observed: *FAT2* (c.6838del, 8.3%), *NIBPL* (c.857G>T, 8.3%), *CDKN1A* (c.122_129del, 8.3%) and APC (c.1875_1878del, 8.3%). A concordance was observed between the five patients’ tumors and their corresponding xenografts (CD3, CD6, CD7, CD8 and CD39); however, these had various discrepancies. Hence, in the CD7 model, the homozygous deletion of *CDKN2A/2B* and the *NIBPL* variant (c.857G>T) was not observed in the primary patient’s tumor. In the CD39 model harboring a *PBRM1* variant (c.599C>G), we observed a different *PBRM1* variant in the primary tumor (c.835dup). Finally, in the CD6 model, we noted that the primary tumor did not harbor the *FAT2* variant (c.6838del) observed in the corresponding xenograft. To avoid any doubt concerning the originating link between the patients’ tumors and PDXs, an identity monitoring confirmed the xenograft tumor matched with the corresponding patient’s tumor, suggesting a possible heterogeneity of the human tumors. The *PBRM1* or *SMARCB1* inactivating pathogenic variants were also tested for their impact on patients’ PFS. No significant correlation was found (*p* = 0.11).

### 3.4. In Vivo Antitumor Efficacy of Targeted Therapies

In our PDX panel, four PDX models harboring a pathogenic variant of the SWI/SNF complexes (CD25, CD36, CD39 and CD41) were considered to be tested with the targeted drug, tazemetostat. A therapeutic experiment was only carried out in the *PBRM1*-mutated CD39 PDX, considering a very slow tumor growth in the others (more than 6 months). This PDX model was obtained from a primary skull base tumor previously operated and defined by a *PBRM1* variant (c.599C>G). For this model, PDX tumor-bearing mice were randomized into treatment and control groups (*n* = 5 mice per group).

**Figure 4:**
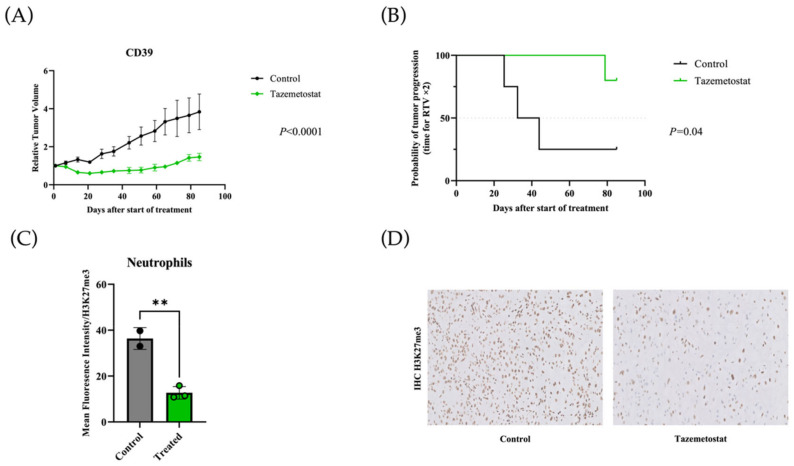
In vivo efficacy of tazemetostat in the CD39 chordoma PDX and pharmacodynamics study. PDX tumor-bearing mice were randomized into each treatment group (*n* = 5 per group) and treated with tazemetostat 75 mg/kg twice a day, 5 days per week (green). Untreated control is shown in black. (**A**) Relative tumor volume. Tumor growth was evaluated by plotting the mean of the relative tumor volume ± SD per group. (**B**) Probability of tumor progression. The time to reach RTV x 2 for each treated mouse was calculated. (**C**) Results for the FACS analysis in neutrophils. A clear shift of the H3K27me3 staining in the treated group compared to the control was observed (** achieve statistical significance compared to control (*p* = 0.005) by Mann–Whitney test). (**D**) Immunohistochemical (IHC) staining results for H3K27me3 in treated mice with anti-EZH2 drug (tazemetostat) and control mice. A decreased expression of H3K27me3 was noted in the treatment group in comparison with the control group (magnification ×20).

Tazemetostat showed a dramatic antitumor efficacy (*p* < 0.0001) with an optimal TGI of 71.5% and an ORR of 100% (Figure 4A). Concerning the probability of tumor progression evaluated by RTV2 analysis (Figure 4B), the tazemetostat treatment appeared to be significantly efficient (*p* = 0.04).

### 3.5. Pharmacodynamics Study

At the end of the tazemetostat treatment, we collected fresh tumors and PBMC to assess the efficacy of the drug on EZH2 activity. With this aim, we first analyzed the relative level of H3K27me3 staining in PBMC by FACS analysis. As shown on Figure 4C, we noticed a clear shift superior to 50% of the H3K27me3 staining after treatment in all nuclear cells, particularly in neutrophils, thus demonstrating the efficacy of the drug (*p* = 0.005). We furthermore confirmed this activity on tumor samples, using similar approaches with H3K27me3 staining; consistently, the staining was significantly decreased in % of the tumor cells/surface (Figure 4D). Altogether, we felt confident that the drug was used at an efficient concentration (FACS plus IHC).

## 4. Discussion

In our work, we first established and characterized the largest panel of chordoma xenografts to date and, secondly, demonstrated a dramatic in vivo effect of the EZH2-inhibitor tazemetostat in one mutated *PBRM1* chordoma PDX model.

Animal models of human cancers, particularly for such a rare tumor as chordoma, as well as the in vivo biological and pharmacological information they can provide, remain critical components in understanding the pathophysiology of the tumor, identifying new drug therapies, and exploring resistance mechanisms to therapies. In the past, very few models as cell lines or xenografts contributed to the study of this rare tumor and to perform pharmacological assessments [24,25,32,33]. Hence, those models remained insufficient for relevant preclinical pharmacologic screenings. In this view, the establishment of a large panel of chordoma models constitutes an essential step for preclinical experiments, particularly when considering the heterogeneity of the tumors, regarding their clinical aspect, prognosis, pathological and genomics features [6,7,30]. By establishing and deeply characterizing several chordoma PDX models from different patients with various clinical features, locations, immunohistochemically and genomic variations, we therefore contribute to a growing arsenal of relevant therapeutic tools in the management of chordomas. Our data demonstrated that xenograft models are histologically and immunohistochemically similar to the original patients’ samples. Moreover, our PDX cohort seems to be representative of the general chordoma patient population with a majority of classic chordomas [4]. The slow growth noted in our study is concordant with the clinical course observed in other models [11,12] and, especially, in human tumors, which may however limit the usefulness of these models. Thirty-eight fresh human chordoma tumors were grafted into nude immunodeficient mice, and 12 tumor grafts were obtained (31.6%) corresponding to a relatively elevated tumor take rate compared to other tumor PDX models, such as breast cancer and melanoma [34,35] (12% and 28%, respectively).

In addition to phenotypic reproducibility, we also observed some differences when comparing genomics between primary patients’ tumors and corresponding xenografts (Table 2), underlining the spatial heterogeneity of this tumor and a possible clonal selection upon transplantation, already reported by some authors [9]. Interestingly, in the CD39 PDX model and the corresponding primary patient’s tumor, we observed different variants affecting the *PBRM1* gene, therefore suggesting the primordial role for *PBRM1* mutations as driver events in chordoma oncogenesis, already reported [7]. In our chordoma PDX panel, homozygous loss of *CDKN2A/2B* was reported in 53.8% of the xenografts, followed by mutations affecting the SWI/SNF complexes (33.3%), representing most of genetic alterations found in chordomas [6,7]. Interestingly, mutations affecting the SWI/SNF complexes were mutually exclusive with homozygous deletion of *CDKN2A/2B* (*p* = 0.008), suggesting that the cell cycle pathway and the epigenetics could be two essential distinct pathways for chordomagenesis. Oddly, we did not find any mutation affecting genes involved in the PI3K signaling pathway in the 12 tumors for which genomic analysis was performed, frequently mutated in chordomas [6] and found in another orthotopic chordoma xenograft model [12]. To date, no data have been reported on a possible favorable prognostic value of PI3K signaling pathway alterations in chordomas, which may possibly explain that all of our PDXs did not present such alterations.

As reported in other cancers [35,36] in which pejorative clinical and biological tumor features are associated with an increased in vivo tumor take, in our study, in vivo tumor take constituted a predictive factor for short progression-free survival (*p* < 0.007) of corresponding originating patients. However, in vivo tumor take was no longer independently predictive of PFS (*p* = 0.34) in our multivariate analysis, possibly due to the limited number of patients. Moreover, the tumor rate was increased when the primary tumor volume was superior to 30 cm^3^ (*p* = 0.04) and in case of dural invasion (*p* = 0.051), criteria which correlated with the prognosis factors described in patients suffering from chordomas [37]. Although a history of previous treatment is also considered as a pejorative prognostic factor in chordomas [37], we did not find a significant correlation between the in vivo tumor take and previous treatment (*p* = 0.16). In brief, because of the high concordance between PDXs and their corresponding patient’s tumor in terms of histopathological and genomic features, we consider that our chordoma xenograft panel constitutes a robust model to test emergent therapies for recurrent chordomas in order to improve the clinical outcome of patients.

As a key epigenetic regulator, the mammalian SWI/SNF chromatin remodeling complex coordinates chromatin compaction and accessibility for gene transcription in an ATP-dependent manner. Mutations in these genes are now described in different types of tumors [38]. Recently, several variants affecting these complexes have been described in two whole genome/exome studies on large chordomas series [6,7] related to skull-base and sacral locations. Mutations affecting *PBRM1*, a specific subunit of the PBAF complex, seem to be potential driver events for chordomas [7]. *PBRM1* mutations are well known in ccRCC, representing the second, after *VHL* mutations, most genetic event described in this cancer [39]. *PBRM1* alterations also represent significant poor prognostic factors for skull-base chordomas [7], as observed in ccRC [40], reinforcing the overlap between these two cancers [41]. In our PDX panel, we did not show a correlation between *PBRM1* alterations and a worse PFS, which might be due to the limited number of PDXs models (*p* = 0.11). The loss of SMARCB1, another main ubiquitous and constant subunit protein of the SWI/SNF complexes, has also been described as a key genetic event in various tumor types, including malignant rhabdoid tumors [42]. In chordomas, the loss of tumor suppression through *SMARCB1*/INI1 inactivation was considered as a new chordoma sub-group (WHO 2020), more frequently affecting the young population and characterized by a poorer prognosis [7,43,44]. These data, as well as our genomic analyses of xenografts (33.3%, *SMARCB1* or *PBRM1*-mutated PDXs), reinforce that epigenetic dysregulation may play an important role in the development of chordoma, especially through driver events affecting *SMARCB1* and *PBRM1* genes. Considering that chordomas are tumors with a high level of recurrence, often unmanageable with local treatment (surgery and/or radiotherapy), developing preclinical models and drugs targeting these frequent alterations is interesting.

**Figure 5:**
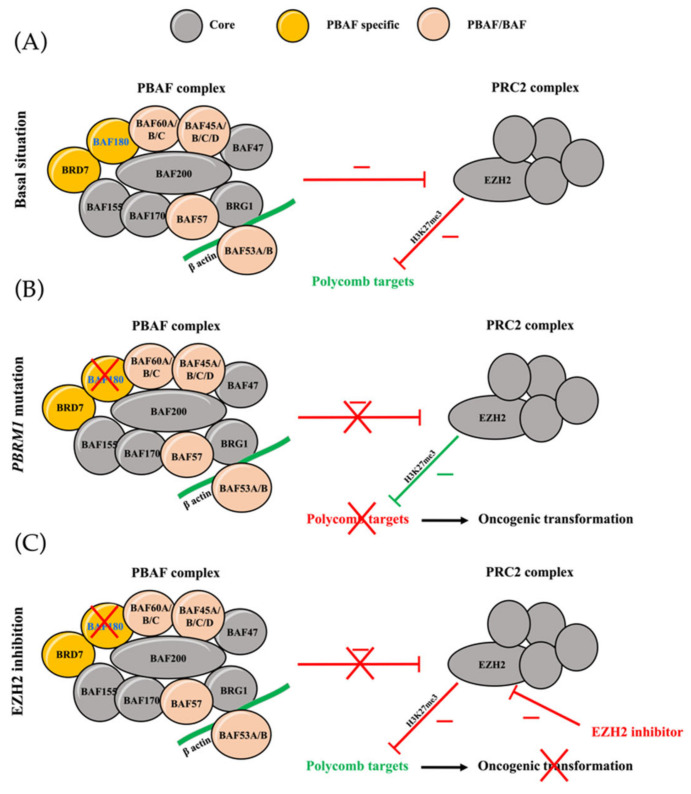
Inter-relationship between PRC2 and SWI/SNF complexes. The SWI/SNF complex is composed of 9 to 12 proteins and characterized by an ATPase function via 2 proteins, SMARCA2 (BRM) and SMARCA4 (BRG) which drives nucleosome remodeling. This complex is formed by a constant protein core and also some optional proteins distinguishing 2 types: BAF (BRG1-associated factors) and PBAF (Polychromo-BRG1-associated factors) complexes. (**A**) In basal situation, SWI/SNF complexes block the PRC2 complex epigenetic silencing of Polycomb targets; in this basal situation, EZH2 acts as the catalytic subunit of the PRC2 Polycomb repressor complex and catalyzes the trimethylation of histone 3 lysine 27 (H3K27me3) at the promoters of target genes. (**B**) Perturbations in SWI/SNF activity, such as the loss of the specific subunit PBRM1 (BAF180), lead to oncogenesis via imbalanced PRC2 activity and aberrant epigenetic silencing of Polycomb targets. (**C**) The EZH2-inhibor re-establishes the balance between the complexes by inhibition of EZH2 methyltransferase activity.

Loss of subunits, such as PBRM1, destabilizes the SWI/SNF complex, resulting in unopposed oncogenic activity of EZH2, an enzymatic catalytic subunit of the PRC2 complex that can alter downstream transcriptions of its target genes involved in cell cycle regulation and cell proliferation by the trimethylation of the lysine 27 of histone 3 (H3K27me3) [27,45] (Figure 5). This observation is found in our study where *PBRM1*-mutated models overexpressed H3K27me3 compared to non-*PBRM1*-mutated models (*p* = 0.05). EZH2 plays a significant role in autophagy, apoptosis, DNA repair and cellular senescence inhibition [27], conferring itself an important role in cancer initiation, progression and metastasis. EZH2 overexpression is also frequently observed in various malignant tumors, such as breast cancer, resulting in a poor prognostic [27]. Therefore, EZH2 becomes a potential hotspot for a tumor-targeted drug, in order to restore the equilibrium between SWI/SNF and PRC2 complexes by inhibition of the EZH2 methyltransferase activity. Promising efficacy has been suggested with EZH2 inhibitors [46], especially in *SMARCB1*/INI1 tumors, including chordomas [47]. Although an efficient antitumoral activity of the anti-EZH2 tazemetostat has already been reported in one *SMARCB1*/INI1 negative chordoma [28], there are no data concerning anti-EZH2 drugs on *PBRM1*-mutated chordoma, which is one of the most common genetic type [7]. In our model harboring a *PBRM1* variant, we observed for the first time a strong and prolonged effect of the EZH2 inhibitor tazemetostat (*p* < 0.0001). This clinical effect was also found in our PD study in which we observed an efficacious activity of the drug via a decreased level of H3K27me3 in FACS (*p* = 0.005) and IHC. Our study supports the hypothesis that *PBRM1*-mutation could be considered as a predictive biomarker of response to EZH2-inhibitors. Hence, this hypothesis might be confirmed through other preclinical, including non-*PBRM1*-mutated models and clinical studies. Moreover, as shown in other human cancer types [27], further study combining EZH2-inhibitors with other treatments, such as immunotherapy or targeted therapies in chordomas, should also be considered to improve the outcome of chordoma patients.

### Study Limitations

First, we did not perform an extensive genomic analysis of all patients’ tumors and their corresponding xenografts and tumors which did not implant into mice. This analysis, by showing a genomic correspondence between the primary patients’ tumors and their corresponding xenografts, may reinforce the robustness of our PDX panel to test innovative drugs. Secondly, we showed a tumor response of tazemetostat in only one model harboring a *PBRM1* variant (out of four models harboring mutations affecting SWI/SNF complexes), considering the slow-growth of the tumor in those PDX models, and we did not test the drug in non-*PBRM1*-mutated models. Further therapeutic experiments on other *PBRM1*-mutated or not models are essential to reinforce the role for *PBRM1* as a biomarker of response to EZH2-inhibitors. Finally, given the fact that our panel does not harbor PI3K/AKT/mTOR mutated models, testing new therapies targeting this pathway was not pertinent.

## 5. Conclusions

The present study described a large panel of chordoma PDXs, corresponding to the clinical outcome, as well as histopathological and genomic features of patients. Given the natural resistance of chordomas to standard chemotherapy, such a panel is mandatory to test novel therapies in advanced chordomas. Moreover, we established for the first time a strong antitumor effect of the EZH2-inhibitor tazemetostat in a *PBRM1*-mutated PDX, suggesting *PBRM1* as a new potential theragnostic marker in chordoma and supporting further evaluation of EZH2-inhibitors in this subgroup of chordomas.

## Figures and Tables

**Table 1:** Clinical and radiological characteristics of all chordoma patients (*n* = 38; univariate analysis) and in vivo tumor take rate (%).

Patients’ Characteristics	Patients (*n*)	Tumor Take Rate (%)	p *
Gender			NS
Male	21	28.6	
Female	17	35.3	
Age at surgery (years)			NS
<50	15	26.7	
≥50	23	34.8	
History of treatment			NS
No	17	21.4	
Yes ^†^	21	42.9	
Primary tumor location			NS
Skull base ^‡^	23	26.1	
No skull base	15	40	
Tumor volume (cm^3^)			**0.03**
<30	20	15	
≥30	18	50	
Intradural invasion ^§^ (*n* = 23)			0.058
No	8	0	
Yes	15	40	
Histology type			NS
Classic	36	29.8	
Chondroid	2	50	
Differentiated	0	0	
Ki-67 (%) (*n* = 37)			**0.001**
<6	18	5.6	
≥6	19	57.9	
Gross total resection			**0.002**
No	14	64.3	
Yes	24	12.5	

^†^ Previous surgery (except single tumor biopsy) and/or radiotherapy. ^‡^ Clival, cranio-cervical junction chordomas (C0–C2). ^§^ Only skull base chordomas. * *p* values were calculated using χ^2^ or Fisher’s test as appropriate.

**Table 2:** Histopathologic and genomic comparison between patients’ tumors and the corresponding xenografts.

PDX Models	Histopathology						Genomic			
	Type		Brachyury		Ki-67 LI ^†^		Variants		CNV Profile	
	P	PDX	P	PDX	P	PDX	P	PDX	P	PDX
CD3	C	C	+	+	≥6	≥6	0	0	del *CDKN2A/CDKN2B*	del *CDKN2A/CDKN2B*
CD6	Ch	Ch	+	+	≥6	≥6	0	*FAT2* (c.6838del; p.(Ser2280ProfsTer12))	0	0
CD7	C	C	+	+	≥6	≥6	0	*NIBPL* (c.857G>T; p.(Gly286Val))	0	del *CDKN2A/CDKN2B*
CD8	C	C	+	+	≥6	≥6	0	0	del *CDKN2A/CDKN2B*	del *CDKN2A/CDKN2B*
CD12	C	C	+	+	≥6	≥6	NA	0	NA	del *CDKN2A/CDKN2B*
CD17	C	C	+	+	≥6	≥6	NA	0	NA	del *CDKN2A/CDKN2B*
CD25	C	C	+	+	≥6	≥6	NA	*CDKN1A* (c.122_129del; p.(Asp41AlafsTer26)) *SMARCB1 (*c.93+2T>G; p?)	NA	0
CD36	C	C	+	+	≥6	≥6	NA	*PBRM1* (c.3222del; p.(Lys1074AsnfsTer85))	NA	0
CD39	C	C	+	*+*	≥6	≥6	*PBRM1* (c.835dup; p.(Ile279AsnfsTer8))	*PBRM1* (c.599C>G; p.(Ser200Ter))	0	0
CD41	C	C	+	+	<6	<6	NA	*PBRM1* (c.2377C>T; p.(Gln793Ter))	NA	0
CD44	C	C	+	+	≥6	≥6	NA	*APC* (c.1875_1878del; p.(Asn627LeufsTer2))	NA	del *CDKN2A/CDKN2B*
CD45	C	C	+	+	≥6	≥6	NA	0	NA	del *CDKN2A/CDKN2B*

Abbreviations: PDX, xenograft obtained from primary tumors; P, patient’s tumor; C, classic histological type according to the WHO classification 2016; Ch, chondroid subtype; NA: not available data. ^†^ Data are expressed as < or ≥6%.

## Data Availability

The data presented in this study are available on request from the corresponding author.
